# Genome Sequence of the Thermophilic Soil Bacterium Ureibacillus terrenus ATCC BAA-384^T^

**DOI:** 10.1128/MRA.01054-21

**Published:** 2021-12-02

**Authors:** Milto Simoes Junior, Kyle S. MacLea

**Affiliations:** a Biotechnology Program, University of New Hampshire, Manchester, New Hampshire, USA; b Biology Program, University of New Hampshire, Manchester, New Hampshire, USA; c Graduate Program in Biotechnology: Industrial and Biomedical Sciences, University of New Hampshire, Manchester, New Hampshire, USA; d Department of Life Sciences, University of New Hampshire, Manchester, New Hampshire, USA; University of Southern California

## Abstract

Ureibacillus terrenus TH9A^T^ (=ATCC BAA-384^T^) was isolated from uncultivated soil in Italy in 1995. We present a draft genome sequence for the type strain, with a predicted genome length of 2,936,851 bp, containing 2,766 protein-coding genes, 82 RNA genes, and 5 CRISPR arrays, with a G+C content of 42.5%.

## ANNOUNCEMENT

Strains from the thermophilic genus *Ureibacillus* have been identified in several different habitats, including compost, soil, landfill and waste treatment systems, and air (1–6). Unusually, *Ureibacillus* spp. are Gram-negative *Firmicutes* that do not grow anaerobically or catabolize sugars as a source of carbon and energy (2). Ureibacillus terrenus TH9A^T^ (=ATCC BAA-384^T^ = DSM 12654^T^ = LMG 19470^T^) was isolated from uncultivated Italian soil in 1995 (1, 2). *U. terrenus* is a rod-shaped, motile species displaying terminal or subterminal spherical endospores under appropriate conditions and is differentiated from the type species, Ureibacillus thermosphaericus, based on its isoprenoid quinone composition and ability to grow at higher temperatures (up to 65°C) and pH (up to 9.0) (2, 3, 5, 7). *U. terrenus* and other members of its genus have been noted as important players in compost and waste decomposition (8–10) and have been investigated for biotechnology applications, given their thermophilic enzymes (11–16). Additionally, given recent emendations of the *Ureibacillus* genus within the family *Caryophanaceae* (17, 18), the completion of the genome sequence for *U. terrenus* will contribute to further discussions of the taxonomic structure of this family.

Freeze-dried *U. terrenus* ATCC BAA-384^T^ cells were obtained from ATCC (Manassas, VA, USA) and then rehydrated in Trypticase soy broth (TSB) and incubated at 55°C for 24 h at 1 atm. After streaking onto Trypticase soy agar, a single colony of *U. terrenus* was grown to log phase at 50°C in 2 ml TSB before its genomic DNA (gDNA) was isolated using the QIAamp DNA minikit (Qiagen, Valencia, CA, USA). gDNA fragmentation and adapter attachment were performed using a KAPA HyperPlus kit v.3.16 (KR1145; Wilmington, MA, USA). Sequencing followed on an Illumina HiSeq 2500 instrument (Hubbard Center for Genome Studies, Durham, NH, USA). Paired-end 250-bp reads were trimmed using Trimmomatic v.0.38 (settings: paired-end mode with a window size of 4, quality requirement of 15, and minimum read length of 36); then, 6,355,970 trimmed reads were assembled using SPAdes v.3.13.0 (19, 20) with default bacterial assembly parameters. Small contigs (<500 bp) were removed, along with any contigs containing contaminants flagged during PGAP (below). QUAST (21) analysis of this assembly showed 80 contigs—the largest 392,574 bp—with an *N*_50_ value of 158,024 bp. A genome coverage of 984× was calculated. The NCBI Prokaryotic Genome Assembly Pipeline (PGAP) (22) was used for gene identification and annotation. The assembled genome was 2,936,851 bp long, and PGAP revealed a total of 2,910 genes, 2,766 protein-coding sequences, 62 pseudogenes, 53 tRNAs, 24 partial or complete copies of the rRNA genes (including 1 complete 16S rRNA gene), 5 noncoding RNAs (ncRNAs), and a G+C content of 42.5%, close to the published values for the species (39.6 to 41.5%) and genus (35.7 to 41.5%) (2). Five CRISPR arrays were identified, as well as the CRISPR-associated genes encoding the enzymes Cas1-3, Cas4a, and Cas6 (23).

### Data availability.

The Ureibacillus terrenus ATCC BAA-384^T^ whole-genome shotgun sequence (WGS) project has been deposited at DDBJ/ENA/GenBank under accession number VIGD00000000. The raw Illumina data were submitted to the NCBI Sequence Read Archive (SRA) under accession number SRX6431131 and BioSample accession number SAMN12147508.
